# Mortality from external causes in late adolescence and early adulthood by gestational age and sex: a population-based cohort study in four Nordic countries

**DOI:** 10.1186/s12916-024-03731-2

**Published:** 2024-11-04

**Authors:** Josephine Funck Bilsteen, Signe Opdahl, Anna Pulakka, Per Ivar Finseth, Weiyao Yin, Kristine Pape, Jorun Schei, Johanna Metsälä, Anne-Marie Nybo Andersen, Sven Sandin, Eero Kajantie, Kari Risnes

**Affiliations:** 1 Department of Clinical and Molecular Medicine, Faculty of Medicine and Health Sciences, Norwegian University of Science and Technology, Trondheim, Norway; 2 Section of Epidemiology, Department of Public Heath, University of Copenhagen, Copenhagen, Denmark; 3 Department of Public Health and Nursing, Faculty of Medicine and Health Sciences, Norwegian University of Science and Technology, Trondheim, Norway; 4 Centre for Big Data Research in Health, University of New South Wales, Kensington, Australia; 5 Population Health Unit, Finnish Institute for Health and Welfare, Helsinki, Finland; 6 Research Unit for Population Health, University of Oulu, Oulu, Finland; 7 Department of Mental Health, Faculty of Medicine and Health Sciences, Norwegian University of Science and Technology, Trondheim, Norway; 8 Division of Mental Health Care, St Olavs Hospital, Trondheim University Hospital, Trondheim, Norway; 9 Department of Medical Epidemiology and Biostatistics, Karolinska Institutet, Stockholm, Sweden; 10 Chief Executive Office, Trondheim Municipality, Trondheim, Norway; 11 Department of Mental Health, Faculty of Medicine and Health Sciences, Regional Centre for Child and Youth Mental Health and Child Welfare, Norwegian University of Science and Technology, Trondheim, Norway; 12 Department of Child and Adolescent Psychiatry, Division of Mental Health Care, St Olavs Hospital, Trondheim University Hospital, Trondheim, Norway; 13 Department of Psychiatry, Icahn School of Medicine at Mount Sinai, New York City, NY USA; 14 Seaver Center for Autism Research and Treatment, Icahn School of Medicine at Mount Sinai, New York, NY USA; 15 Clinical Medicine Research Unit, Oulu University Hospital and University of Oulu, Oulu, Finland; 16 Children’s Clinic, St Olavs Hospital, Trondheim University Hospital, Trondheim, Norway

**Keywords:** Mortality, External causes of death, Suicide, Transport Accidents, Drugs, Alcohol, Gestational age, Preterm birth, Health Registry, Sex differences

## Abstract

**Background:**

External causes of death, such as accidents, substance use, and suicide, contribute substantially to mortality during adolescence and early adulthood and show marked sex differences. Individuals born preterm are at increased risk of mental disorders, and impaired cognitive and executive functions, potentially increasing their vulnerability to death from external causes. We investigated sex-specific associations between gestational age at birth and mortality from external causes during late adolescence and early adulthood.

**Methods:**

Individual level data from national health registries in Denmark (1978–2001), Finland (1987–2003), Norway (1967–2002), and Sweden (1974–2001) were linked to form nationwide cohorts. In total, 6,924,697 participants were followed from age 15 years to a maximum of 50 years in 2016–2018. Gestational age was categorized as “very/moderately preterm” (23–33 weeks), “late preterm” (34–36 weeks), “early term” (37–38 weeks), “full term” (39–41 weeks), and “post term” (42–44 weeks). Outcomes were mortality from external causes overall and from the largest subgroups transport accidents, suicide, and drugs or alcohol. We estimated sex-specific hazard ratios (HRs), with full term as the reference, and pooled each country’s estimates in meta-analyses.

**Results:**

Across gestational ages mortality was higher for males than females. Individuals born very/moderately preterm had higher mortality from external causes, with HRs 1.11 (95% confidence interval [CI] 0.99–1.24) for males and 1.55 (95% CI 1.28–1.88) for females. Corresponding estimates for late preterm born were 1.11 (95% CI 1.04–1.18) and 1.15 (95% CI 1.02–1.29), respectively. Those born very/moderately preterm had higher mortality from transport accidents, but precision was low. For females, suicide mortality was higher following very/moderately preterm birth (HR 1.76, 95% CI 1.34–2.32), but not for males. Mortality from drugs or alcohol was higher in very/moderately and late preterm born males (HRs 1.23 [95% CI 0.99–1.53] and 1.29 [95% CI 1.16–1.45], respectively) and females (HRs 1.53 [95% CI 0.97–2.41] and 1.35 [95% CI 1.07–1.71], respectively, with some heterogeneity across countries).

**Conclusions:**

Mortality from external causes overall was higher in preterm than full term born among both males and females. A clear sex difference was seen for suicide, where preterm birth was a risk factor in females, but not in males.

**Supplementary Information:**

The online version contains supplementary material available at 10.1186/s12916-024-03731-2.

## Background

External causes of death, such as accidents, suicides, substance use and intoxication, are theoretically largely preventable but nevertheless remain major causes of death during adolescence and early adulthood [1, 2]. Population level mortality from external causes is strongly influenced by societal characteristics [1, 3] and is around three times higher in males than in females, thereby accounting for a large proportion of male excess mortality in adolescence and early adulthood [1]. However, an individual’s vulnerability is also influences by factors such as risk-taking behavior, drug use, and mental and neurologic disorders [4, 5], which have been linked to early life development and adversity, including the perinatal period [6–9].

Preterm birth is a common and severe perinatal complication, and compared to term born, individuals who survive preterm birth are more likely to show characteristics and behavior associated with either smaller or larger vulnerability to death from external causes: Some studies suggest individuals born preterm take less risk, indicated by lower occurrence of criminal convictions [10–12], sexually transmitted diseases [10], and teenage pregnancies [10], whereas studies on substance abuse show conflicting results [10–12]. In contrast, those born preterm have higher occurrences of risk factors such as poor cognitive and executive functions [13, 14] and neurodevelopmental and mental disorders such as attention-deficit/hyperactivity disorder (ADHD) [15], autism spectrum disorders (ASD) [16], psychosis, depression, and bipolar and eating disorders [12, 17, 18]. Moreover, risk of impaired neurodevelopmental and mental health is highest for those born at the lowest gestational ages [16, 19].

Preterm birth is more common in pregnancies with a male fetus, and neonatal mortality and morbidity after preterm birth is also higher among males [20–22]. However, it is not clear whether these sex differences in neonatal outcomes translate into systematic sex differences in subsequent neurodevelopmental and mental health [16, 18, 23]. One possible exception is that there are indications that lower gestational age is associated with emotional problems in early childhood [24] and depression in youth [25] for females, but not for males. These observations are also supported by a steeper increase in use of antidepressants and other psychotropic drugs through adolescence and early adulthood for females born preterm than preterm born males and term born females [26].

Modern medical care has increased neonatal survival after preterm birth substantially across the latest decades [27]. As a result, there is a continuous need to assess long-term health and life expectancy among survivors, many of whom are currently young adults. Adults who were born preterm have higher all-cause mortality than those born at term, some of which may be attributed to a higher mortality from chronic diseases [28, 29]. In previous Norwegian [30] and Swedish [29] studies, preterm birth was associated with higher mortality during adolescence and adulthood from all external causes combined. For risk of suicide, a recent meta-analysis of five cohorts of adolescents and adults [7], including estimates from Norway [30] and Sweden [31], indicated modestly higher risk for individuals born preterm, but confidence intervals were wide. Subsequent analyses of Swedish data supported these findings in adult females, but not in adult males [32]. For other external causes of death, such as accidents and substance use, evidence is even more limited, with a single study indicating higher risk in adolescents and adult born preterm [30].

Understanding whether the association between preterm birth and external causes of death is limited to specific causes, and whether the risk varies across different severities of preterm birth or between males and females, will help establish the long-term consequences of preterm birth and may also provide opportunities for their prevention. Our aim was to investigate the association between gestational age at birth and mortality from external causes during late adolescence and adulthood, overall and from specific causes, i.e., suicide, transport accidents, and deaths from drug and alcohol. We did this using nationwide, individual-level registry data from four Nordic countries, with separate analyses for males and females.

## Methods

Individual level data from nationwide Medical Birth Registries (MBRs) in Denmark, Finland, Norway, and Sweden, were linked to other nationwide data using national identity numbers assigned to all residents at birth or immigration [33].

### Study populations

We identified 7,389,282 liveborn individuals in the Danish (1978–2001), Finnish (1987–2003), Norwegian (1967–2002), and Swedish (1974–2001) MBR [34]. Of these, 7,178,219 (97.1%) were alive and resident at age 15 years (start of follow-up), which is the age when mortality from external causes has been estimated to start increasing [35]. Maximum follow-up was until 50 years in the oldest birth cohort. We excluded 224,703 (3.1%) individuals with missing data on gestational age, birthweight, sex, birth order, and maternal age, 8857 (0.1%) with gestational age outside the range 23–44 completed weeks, and 19,812 (0.3%) with unlikely combinations of birthweight and gestational age (< − 6/ > + 4 standard deviations from expected fetal weight by gestational age and sex, as estimated by Maršál and colleagues [36]). Country-specific number of exclusions are presented in Additional file 1: Fig. S1.

### Exposure

Gestational age in days or completed weeks was obtained from the MBRs. Estimation by ultrasound examination was preferred over last menstrual period if both were available. For deliveries after assisted reproduction, estimation using date of embryo transfer was preferred. Gestational age was categorized as “very/moderately preterm” (23–33 weeks), “late preterm” (34–36 weeks), “early term” (37–38 weeks), “full term” (39–41 weeks), and “post term” (42–44 weeks).

### Outcomes

Causes of death were available from each country’s national Cause of Death Registry and coded according to the International Statistical Classification of Diseases and Related Health Problems (ICD), versions 8–10. Outcomes were external causes of death overall and the three largest subgroups of external causes, namely fatal transport accidents, suicides, and deaths from drugs or alcohol, defined according to the 2012 European Shortlist for Causes of Death and the Nordic Medico-Statistical Committee [35]. ICD codes included in the definitions of all outcomes are listed in Additional file 1: Table S1.

### Covariates

Information on birth year, sex, birth order, and maternal age at birth was obtained from MBRs and categorized as shown in Table 1. From national education databases, we obtained information on maternal and paternal educational level in the child’s birth year, as an indicator of socioeconomic position. Education data were harmonized according to the International Standard Classification of Education (ISCED) version 11 and combined into highest parental educational level: lower (ISCED 0–2), intermediate (ISCED 3–5), higher (ISCED 6–8), and missing (ISCED 9, if missing for both parents). For Sweden, maternal education was available from 1990 onwards and was used instead of parental education because paternal education was not available. History of parental suicide was defined as a parent (in Sweden only the mother) being registered with suicide as cause of death, at any time during the study period. For participants with unknown father, measures of parental education and history of suicide was based on maternal data only.

**Table 1:** Characteristics of the Danish, Finnish, Norwegian, and Swedish study populations

Cohort characteristics	Denmark	Finland	Norway	Sweden
**Participants, *n***	1,360,453	1,006,583	1,890,826	2,666,835
**Median follow-up in years [IQR]**	10.6 [5.5–16.5]	8.8 [4.5–12.8]	17.6 [8.5–27.7]	12.9 [7.3–20.2]
**Median age at end of follow-up in years [IQR]**	25.6 [20.5–31.5]	23.8 [19.6–27.8]	32.6 [23.5–42.7]	27.9 [22.3–35.2]
**Gestational age, *n* (%)**				
23–33 weeks	18,984 (1.4)	12,857 (1.3)	23,693 (1.3)	33,768 (1.3)
34–36 weeks	52,973 (3.9)	39,803 (4.0)	74,804 (4.0)	113,610 (4.3)
37–38 weeks	196,422 (14.4)	180,586 (17.9)	252,106 (13.3)	473,163 (17.7)
39–41 weeks	972,586 (71.5)	728,850 (72.4)	1,291,302 (68.3)	1,820,753 (68.3)
42–44 weeks	119,488 (8.8)	44,487 (4.4)	248,921 (13.2)	225,541 (8.5)
**Birth year, *n* (%)**				
1967–1974	-	-	478,468 (25.3)	103,469 (3.9)
1975–1979	84,244 (6.2)	-	243,620 (12.9)	458,872 (17.2)
1980–1984	240,570 (17.7)	-	228,954 (12.1)	437,659 (16.4)
1985–1989	278,174 (20.4)	180,265 (17.9)	241,622 (12.8)	504,941 (18.9)
1990–1994	320,500 (23.6)	317,581 (31.6)	265,656 (14.0)	562,448 (21.1)
1995–2003	436,965 (32.1)	508,737 (50.5)	432,506 (22.9)	599,446 (22.5)
**Females, *n* (%)**	662,824 (48.7)	492,610 (48.9)	920,886 (48.7)	1,297,244 (48.6)
**Multiples, *n* (%)**	36,903 (2.7)	27,677 (2.8)	44,756 (2.4)	59,941 (2.2)
**Congenital anomalies, *n* (%)**	27,690 (2.0)	32,967 (3.3)	38,794 (2.1)	108,473 (4.1)
**Median birthweight, grams [IQR]**	3470 [3100–3800]	3570 [3230–3900]	3540 [3200–3880]	3530 [3180–3870]
**Birth order**				
Firstborn	538,916 (39.6)	405,052 (40.2)	780,847 (41.3)	1,097,304 (41.1)
Second or later	821,537 (60.4)	601,531 (59.8)	1,109,979 (58.7)	1,569,531 (58.9)
**Maternal age, *n* (%)**				
≤ 24 years	333,881 (24.5)	203,765 (20.2)	644,882 (34.1)	694,857 (26.1)
25–29 years	532,650 (39.2)	350,933 (34.9)	661,006 (35.0)	984,316 (36.9)
30–34 years	359,366 (26.4)	293,630 (29.2)	409,167 (21.6)	686,223 (25.7)
≥ 35 years	134,556 (9.9)	158,255 (15.7)	175,771 (9.3)	301,439 (11.3)
**Maternal education**^**a**^**, *n* (%)**				
Lower (ISCED 0–2)	448,259 (32.9)	176,978 (17.6)	525,822 (27.8)	351,023 (13.2)
Intermediate (ISCED 3–5)	596,608 (43.9)	678,174 (67.4)	761,001 (40.2)	944,548 (35.4)
Higher (ISCED 6–8)	274,758 (20.2)	146,618 (14.6)	391,777 (20.7)	183,736 (6.9)
Missing	40,828 (3.0)	4813 (0.5)	212,226 (11.2)	1,187,528 (44.5)
**Paternal education**^**a**^**, *n* (%)**				
Lower (ISCED 0–2)	340,906 (25.1)	214,422 (21.3)	449,989 (23.8)	NA
Intermediate (ISCED 3–5)	712,886 (52.4)	613,715 (61.0)	823,181 (43.5)	NA
Higher (ISCED 6–8)	222,229 (16.3)	162,867 (16.2)	398,910 (21.1)	NA
Missing	84,432 (6.2)	15,579 (1.6)	218,746 (11.6)	NA
**Parental education**^**a**^**, *n* (%)**				
Lower (ISCED 0–2)	233,229 (17.1)	77,617 (7.7)	256,744 (13.6)	NA
Intermediate (ISCED 3–5)	742,465 (54.6)	688,999 (68.5)	882,779 (46.7)	NA
Higher (ISCED 6–8)	372,109 (27.4)	236,569 (23.5)	560,173 (29.6)	NA
Missing	12,650 (0.9)	3398 (0.3)	191,130 (10.1)	NA

*IQR*, interquartile range; *ISCED*, International Standard Classification of Education; *NA*, not available

^a^In the Finnish study population, “lower education” included individuals whose parents were registered without an ISCED-level in Statistics Finland’s education registry, where ISCED levels below 3 are not registered. Missing parental education was defined as parents not registered in Statistics Finland’s education registry

### Statistical analyses

Participants were followed from age 15 years to death, emigration, or end of follow-up (Denmark and Sweden: 31/12/2016, Finland: 31/12/2018, Norway: 31/12/2017), whichever came first. We used Cox regression to estimate cause-specific hazard ratios (HR) with 95% confidence intervals (CIs) according to gestational age categories, censoring at death from competing causes. Age during follow-up was the time scale in all analyses. Due to sex differences in neonatal outcomes after preterm birth [20–22] and in mortality from external causes in the general population [1, 35], we examined whether associations with gestational age differed for males and females by including interaction terms between sex and gestational age categories. All outcomes except fatal transport accidents showed interactions in at least one country (*p* < 0.05), and consequently, we estimated sex-specific associations. In each country, adjusted HRs were estimated for combinations of gestational age and sex with a minimum of five deaths. We adjusted for the available confounders birth year, birth order, maternal age, and parental education, defined a priori based on literature and causal framework [37]. Missing data were not imputed due to low proportions of missingness, or, in the case of educational data from Sweden, due to lack of good predictors of educational level before 1990. Proportionality of hazards was examined by visual inspection, with no clear violations found. To account for multiples, we included a pregnancy identifier in the models and computed robust sandwich covariance matrix estimates [38]. Data from Norway and Sweden, but not from Denmark and Finland, could be pooled at an individual level. Hence, analyses were performed for each country separately, following the same syntax. Estimates from each country were combined in a fixed effects meta-analysis using the inverse-variance method for each comparison [39, 40].

To investigate the robustness of the results, we repeated analyses with (1) inclusion of deaths with unknown intent in analyses of suicide, to account for potential misclassification [41, 42]; (2) exclusion of legal drugs in analyses of deaths from drugs or alcohol, to account for the fact that individuals born preterm might need medications with a potential for substance abuse and overdose more often than term born; (3) adjustment for parental history of suicide as a marker of parental severe mental illness [43], associated with offspring preterm birth [44] and external causes of death or related events [45, 46]; and (4) restriction to individuals born 1987 (when the Finnish MBR was established) or later, to ensure similar age and missingness distributions across countries.

## Results

The study population included 6,924,697 individuals, and 370,492 of these were born preterm (< 37 weeks), comprising 5.3–5.6% of the study population in each country. Risk of preterm birth was similar across countries (1.3–1.4% were born very/moderately preterm, and 3.9–4.3% were born late preterm (Table 1)). The countries’ study populations had a similar proportion of females (48.6–48.9%), and differences in birth order were small (39.6–41.3% were firstborn). Maternal age was lower in Norway, where 25.3% of the study population was born before 1975. In Finland and Sweden, intermediate maternal education level was relatively more common than in Denmark and Norway. During follow-up 29,758 participants (0.43%) died from external causes overall, and among these, 7850 (26.4%) died from transport accidents, 11,320 (38.0%) from suicide, and 7785 (26.2%) from drugs or alcohol. Corresponding numbers of death among preterm born were 1724 overall (0.47%), with 440 (25.5%), 615 (35.7%), and 499 (28.9%) for each subgroup, respectively.

**Fig. 1:**
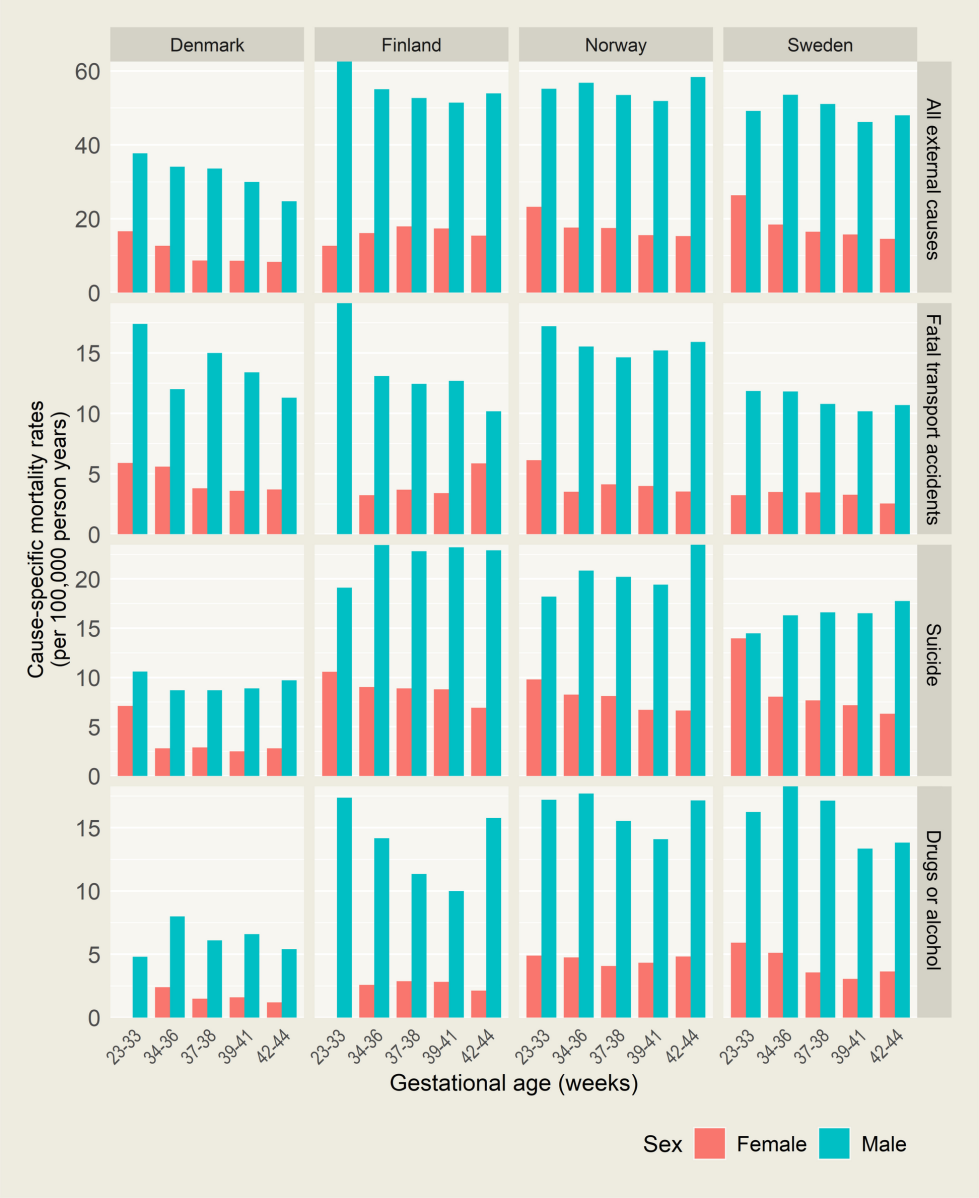
Unadjusted rates of death from external causes according to country, sex, and gestational age at birth. Follow-up from age 15 years to a maximum of 50 years, among individuals born 1978–2001 in Denmark, 1987–2003 in Finland, 1967–2002 in Norway, and 1974–2001 in Sweden. Note that the different outcomes have different y-scales. Categories with no rates have either no events or are censored to maintain data privacy

**Fig. 2:**
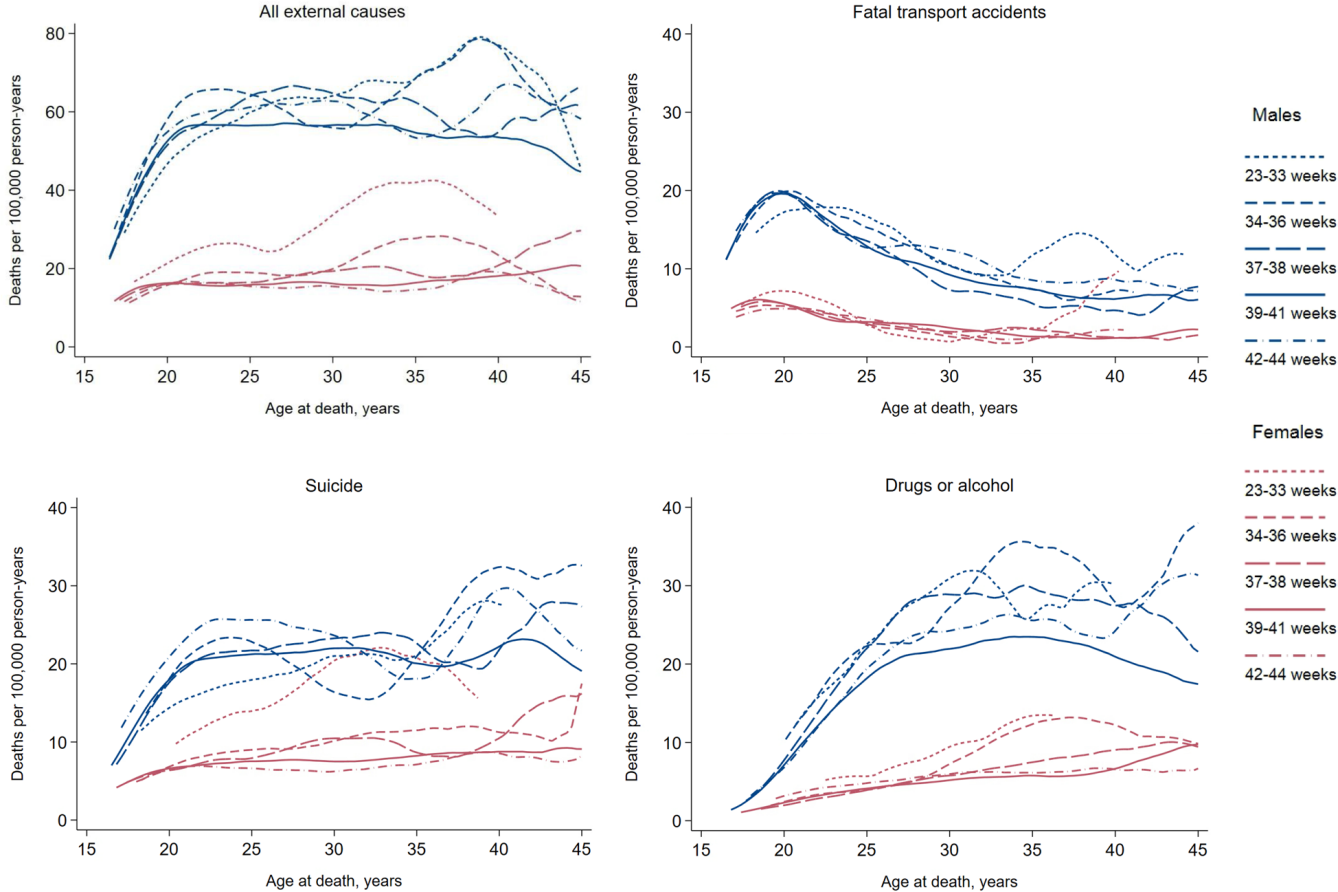
Smoothed, unadjusted hazard rates of external causes of death according to age during follow-up, sex, and gestational age at birth. Individuals born in Norway (1967–2002) and Sweden (1974–2001). The hazard function is based on a weighted kernel-density estimate (Epanechnikov), using the bandwidth that would minimize the mean integrated squared error if the data were Gaussian and a Gaussian kernel were used. For each category, the plot range is constrained to the earliest failure time plus the bandwidth, and the latest failure time minus the bandwidth. Note that the different outcomes have different y-scales

Across all countries and gestational ages, males had higher unadjusted mortality from external causes than females, overall and for each subgroup of causes (Fig. 1). For all outcomes, rates increased steeply with age during late adolescence (Fig. 2). During adulthood, rates levelled off for external causes overall and for suicide but declined gradually for transport accidents. Mortality from drugs or alcohol continued to increase with age among females but declined somewhat from around age 30 years among males.

**Fig. 3:**
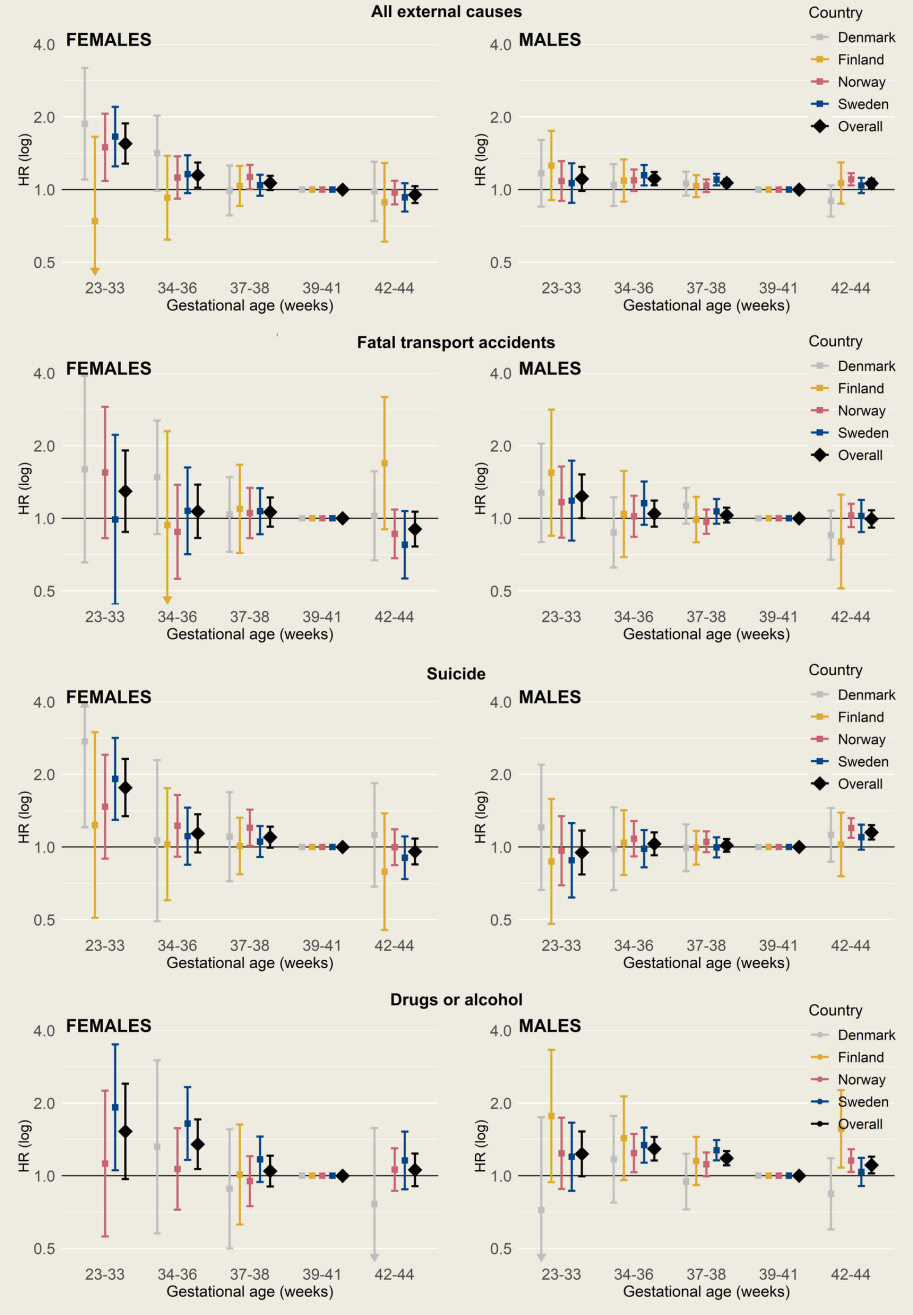
Adjusted hazard ratios (HRs) of external causes of death according to country, sex, and gestational age at birth. Adjusted for age, birth year, birth order, maternal age, and highest parental education at birth. For Swedish data, maternal education at birth was used as paternal education was not available. HRs (squares or diamonds) with 95% confidence intervals (error bands). HRs for gestational age and sex categories with < 5 deaths are not shown. For confidence intervals expanding the range 0.44–4.0, the full interval is not shown, as indicated by arrows. All estimates can be found in Additional file 1: Tables S2–S5. Note that the y-scales are logarithmic

When adjusted estimates were pooled across countries, those born preterm had higher mortality from external causes overall, compared with those born full term (Fig. 3, with corresponding numbers in Additional file 1: Table S2). For males born very/moderately preterm, adjusted HR was 1.11 (95% CI 0.99–1.24), and for females, it was 1.55 (95% CI 1.28–1.88). Corresponding estimates for those born late preterm were 1.11 (95% CI 1.04–1.18) for males and 1.15 (95% CI 1.02–1.29) for females. Those born early term also had slightly higher mortality from external causes (HR 1.07 [95% CI 1.03–1.10] for males and 1.06 [95% CI 1.00–1.14] for females). Point estimates were in the same direction for males in all countries, but for females, no clear association was found in Finland. Being born post term was not associated with mortality from external causes in females, whereas males born post term had slightly higher mortality from external causes overall (HR 1.06, 95% CI 1.02–1.11).

There were indications that being born very/moderately preterm was associated with higher mortality from transport accidents (Fig. 3, with corresponding numbers in Additional file 1: Table S3). However, precision was low, and although point estimates were relatively consistent across countries for males, they fluctuated for females.

Preterm born females had a higher risk of suicide than females born full term (Fig. 3), with those born very/moderately preterm carrying the highest risk (HR 1.76, 95% CI 1.34–2.32, see Additional file 1: Table S4) and age-specific rates approaching those seen among males (Fig. 2). Females born late preterm and early term also had modestly higher rates of suicide, with pooled HRs of 1.14 (95% CI 0.95–1.37) and 1.10 (95% 0.99–1.21), respectively, although CIs were also compatible with no association. Patterns were similar in Denmark, Norway, and Sweden, while no association was observed in Finland. Among males, no association between preterm birth and suicide was observed, whereas males born post term were at higher risk than their term born peers (HR 1.15, 95% CI 1.07–1.23). Findings remained similar when deaths of unknown intent were included in the definition of suicide (see Additional file 1: Tables S6-S7).

Preterm birth was associated with higher mortality from drugs or alcohol (Fig. 3, with corresponding numbers in Additional file 1: Table S5). For very/moderately preterm born, pooled estimates were higher compared to full term, but CIs were compatible with a range from null to substantial associations (HR 1.23 [95% CI 0.99–1.53] for males and 1.53 [95% CI 0.97–2.41] for females). Corresponding estimates for males and females born late preterm were 1.29 (95% CI 1.16–1.45) and 1.35 (95% CI 1.07–1.71), respectively. There was heterogeneity across countries, with estimates in the same direction for males in all countries except Denmark and the combined estimates for females driven by a strong association in Sweden, whereas no association was found in Denmark and Norway. Associations remained similar when restricting the outcome to deaths from illegal drugs or alcohol (see Additional file 1: Table S2 and Fig. S2).

For all outcomes, there were only minor differences between unadjusted and adjusted estimates (see Additional file 1: Tables S2-S5). Adjustment for parental history of suicide did not influence the results (see Additional file 1, Fig. S3). In sensitivity analyses with restriction to individuals born in 1987 or later, i.e., with similar age distributions across countries, absolute mortality in the younger Danish, Norwegian, and Swedish populations was lower than in the main sample, and Finland had the highest absolute mortality (see Additional file 1: Fig. S4). The higher risk of suicide for females persisted only in very/moderately preterm born Norwegians (see Additional file 1: Fig. S5), whose absolute mortality from suicide was higher than for males of any gestational age. The tendency towards higher mortality from transport accidents with lower gestational age among males, and results for mortality from drugs or alcohol remained similar for all countries combined.

## Discussion

Across four Nordic countries, adolescents and adults born preterm had higher sex-specific mortality from external causes overall than peers born full term. Males had higher mortality than females at all gestational ages. When further categorizing external causes of death, the clearest sex differences were seen for mortality from suicide, with a strong association with preterm birth among females and no association among males. For both males and females, being born preterm was associated with higher mortality from drugs or alcohol, and from transport accidents, but with wider confidence intervals and more heterogeneity among females.

Our results are consistent with previous Nordic registry studies indicating higher mortality from external causes overall [29, 30], as well as from suicide [30, 32] and drugs and alcohol [30], for preterm born. A recent meta-analysis, without sex-specific estimates, showed modestly higher suicide mortality after preterm birth, with a total of 371 suicides among preterm born [7]. However, higher suicide mortality for females born preterm has been reported in Sweden, although the association attenuated in comparisons with term born siblings (male and female siblings combined) [32]. Our study strengthens the evidence of this female-specific association by demonstrating consistency across Denmark, Norway, and Sweden. Studies on mortality from accidents and drugs or alcohol among preterm born are limited [30]. However, there are some indications that being born preterm is associated with higher hospitalization rates from injuries and accidents [47–49], whereas previous studies on hospitalization for substance use show inconsistent results in preterm born [10, 12, 50, 51].

Individuals born preterm, particularly those born very preterm (< 28 weeks), more often than term born show behavior referred to as “the preterm behavioral phenotype,” characterized by inattention, anxiety, and social problems through childhood, adolescence, and early adulthood [52–55]. Longitudinal studies of child development indicate that a higher risk of suicide attempts in adolescents exposed to perinatal adversity, including poor fetal growth, may in part be mediated by emotional and behavioral problems, victimization, lower cognitive skills, and in particular hyperactivity-impulsivity [8]. A recent meta-analysis of clinical cohorts [18], and several Nordic registry-based studies [16, 17, 51], show higher occurrence of autism spectrum disorders, ADHD, anxiety and mood disorders, and non-affective psychosis, among individuals born very preterm. Furthermore, preterm born often have symptoms of psychiatric syndromes without meeting all diagnostic criteria [52, 55–57]. Mental illness is a major risk factor for suicide [43] and accidental death [58] and often co-occur with addiction [59]. For example, a diagnosis of ADHD is associated with higher mortality from external causes, particularly if comorbidity from behavioral and/or substance use disorder is present [5]. Higher mortality from drugs or alcohol among those born preterm might result from higher consumption, from increased biological and psychosocial vulnerability, or secondary to pharmacological treatment of organic sequelae such as spasms, pain, seizures, and chronic diseases. However, the fact that results remained similar when restricting the outcome to illegal drugs or alcohol indicate that associations were not driven by deaths from prescribed drugs.

Sex differences in association between gestational age and mortality were most consistent for suicide, but the mechanisms behind this finding are unknown. At the population level, suicide attempts are more common in females, but males often use more violent methods and have higher mortality from suicide [60]. There are indications that suicide attempts are more common in preterm born, but it is not known whether this applies specifically to females [12]. Furthermore, no clear sex differences in associations of preterm birth with presumed intermediates such as psychiatric diagnoses or neurodevelopmental outcomes have been reported [17, 18, 23]. However, analyses of Norwegian registry data indicate use of psychotropic drugs may increase more steeply from adolescence to early adulthood among females born preterm than among males and term-born females [26], possibly indicating more severe or accelerating symptoms in this group.

Improved understanding of events leading to deaths from external causes among preterm born may provide opportunities for prevention, despite the challenges in preventing preterm birth or its direct long-term sequels. Awareness of higher vulnerability among the preterm born themselves, their parents, school staff, and clinicians could help avoid high-risk situations such as drug or alcohol problems, bereavement, or suicidal ideation or improve their management [3]. Importantly, age-specific rates gave no clear indications that this vulnerability was limited to specific ages, although precision was low.

## Strengths and limitations

A key strength of the study is the inclusion of nationwide data from four Nordic countries, resulting in a uniquely large study population of almost 7 million individuals and a maximum follow-up to age 50 years. Furthermore, data were harmonized, and identical analytical approaches applied in all four countries, facilitating comparison and pooling of estimates in meta-analysis, with reasonable precision in most analyses. The Nordic countries have comparable, although not identical, health care systems and prenatal care programs and similar health registries [33–35]. The occurrence of preterm birth has been relatively stable in all four countries during the study period [61]. The countries also show similarly declining trends in mortality from transport accidents and suicide at the population level [35], whereas trends in mortality from drugs or alcohol are less uniform [62, 63].

Parental psychiatric disorders are associated with preterm birth [44, 64], and several psychiatric disorders associated with suicidal behavior show high heritability [45]. Although adjustment for parental suicide, a marker of parental mental health, gave similar results as the main models, residual confounding from parental mental health or from other family factors cannot be excluded. Changes over time in methods for estimation of gestational age may have introduced misclassification [65], and combined with relatively broad categories of gestational age, this might mask stronger associations for those born at the lowest gestational ages.

Subclassification of external causes of death is challenging because suicidal motives may be difficult to disentangle from accidents, whether through poisoning/overdoses or physical accidents [66]. This is reflected in validation of data in the Cause of Death Registries in Denmark, Norway, and Sweden, showing 90%, 81%, and 88% agreement, respectively, between the registered cause of death and an expert panel for suicide [41], and somewhat lower for accidents [42]. To address concerns on misclassification of suicides, we compared findings with and without inclusion of deaths with unknown intent in the suicide definition, which gave similar results. Some differences in coding practices across countries are also expected based on national traditions and adaptations of the ICD system [35]. However, since 2001, all four countries have implemented the Automated Classification of Medical Entities, which increases consistency and thereby comparability [35].

During our study period, neonatal and childhood survival increased substantially for individuals born very/moderately preterm, and an increasing proportion of severely ill preterm born children have reached adulthood [67–71]. Although this development may have introduced survivor bias in our estimates, the consistency in associations when restricting the population to those born 1987 or later suggests that the associations were not driven by period effects in survival up to 15 years. Furthermore, there are indications that improvements in neonatal morbidity and mortality after preterm birth have not been accompanied by improvements in cognitive or other long-term outcomes [72–75]. This emphasizes the need for continued follow-up, also including more recent birth cohorts exposed to contemporary management of both preterm and post term delivery [27], to clarify the long-term relevance and generalizability of our findings.

## Conclusions

In this meta-analysis of nationwide Nordic cohorts, being born preterm was associated with higher sex-specific mortality from external causes during late adolescence and early adulthood compared to being born full term. For males, who had a higher absolute mortality from all external causes than females across gestational age, the association with preterm birth was modest and driven by higher mortality from drugs or alcohol and to some extent from transport accidents. For females, the association with preterm birth was stronger than for males and this difference was primarily attributed to a higher mortality from suicide. Further investigations are needed to assess how preventive strategies can meet the needs of those born preterm.

## Supplementary Information

Additional file 1. Fig. S1. Flow chart showing inclusions and exclusions from the study population in each country. Table S1. Overview of ICD versions and codes used to define outcomes, and when these were used in each country. Table S2. Unadjusted and adjusted associations of gestational age at birth with mortality from all external causes during late adolescence and adulthood, by sex and country of birth. Table S3. Unadjusted and adjusted associations of gestational age at birth with mortality from transport accidents during late adolescence and adulthood, by sex and country of birth. Table S4. Unadjusted and adjusted associations of gestational age at birth with mortality from suicide during late adolescence and adulthood, by sex and country of birth. Table S5. Unadjusted and adjusted associations of gestational age at birth with mortality from drugs or alcohol during late adolescence and adulthood, by sex and country of birth. Table S6. Adjusted associations of gestational age with mortality from external causes, according to sex and country of birth. Sensitivity analyses using other outcome definitions for suicide and death from drugs or alcohol. Fig. S2. Adjusted hazard ratios (HRs) of suicide and death from drugs or alcohol according to country, sex, and gestational age at birth. Sensitivity analyses using alternative outcome definitions. Fig. S3. Adjusted hazard ratios (HRs) of external causes of death according to country, sex, and gestational age at birth. Sensitivity analyses with additional adjustment for family history of suicide. Fig. S4. Unadjusted rates of death from external causes according to country, sex, and gestational age at birth. Sensitivity analysis in populations born 1987 or later. Fig. S5. Adjusted hazard ratios (HRs) of external causes of death according to country, sex, and gestational age at birth. Sensitivity analysis in populations born 1987 or later.

## Data Availability

The data that support the findings of this study are available from the registry-keeping authorities in Denmark, Finland, Norway, and Sweden, but national laws restrict the availability of these data, which were used under license for the current study, and so are not publicly available. Data access requires ethics approvals as stated above and approvals from the registry-keeping authorities and the responsible research institutions in each country.

## Abbreviations

ADHD: Attention-deficit/hyperactivity disorder
ASD: Autism spectrum disorder
CI: Confidence interval
HR: Hazard ratio
ICD: International Statistical Classification of Diseases and Related Health Problems
ISCED: International Standard Classification of Education
MBR: Medical Birth Registry
RECAP: Research on Children and Adults Born Preterm
