# Supplementary material for: Mortality from external causes in late adolescence and early adulthood by gestational age and sex: a population-based cohort study in four Nordic countries
Source: BMC Med. 2024 Nov 4;22:506. doi: 10.1186/s12916-024-03731-2 (PMC11536539; doi:10.1186/s12916-024-03731-2)

Additional file 1

Mortality from external causes in late adolescence and early adulthood by gestational age and sex: a population-based cohort study in four Nordic countries

Josephine Funck Bilsteen, Signe Opdahl, Anna Pulakka, Per Ivar Finseth, Weiyao Yin, Kristine Pape, Jorun Schei, Johanna Metsälä, Anne-Marie Nybo Andersen, Sven Sandin, Eero Kajantie, Kari Risnes

Content

Figure S1. Flow chart showing inclusions and exclusions from the study population in each country. ... 2

Table S1. Overview of ICD versions and codes used to define outcomes, and when these were used in each country. ... 3

Table S2. Unadjusted and adjusted associations of gestational age at birth with mortality from all external causes during late adolescence and adulthood, by sex and country of birth. ... 4

Table S3. Unadjusted and adjusted associations of gestational age at birth with mortality from transport accidents during late adolescence and adulthood, by sex and country of birth. ... 5

Table S4. Unadjusted and adjusted associations of gestational age at birth with mortality from suicide during late adolescence and adulthood, by sex and country of birth. ... 6

Table S5. Unadjusted and adjusted associations of gestational age at birth with mortality from drugs or alcohol during late adolescence and adulthood, by sex and country of birth. ... 7

Table S6. Adjusted associations of gestational age with mortality from external causes, according to sex and country of birth. Sensitivity analyses using other outcome definitions for suicide and death from drugs or alcohol. ... 8

Figure S2. Adjusted hazard ratios (HRs) of suicide and death from drugs or alcohol according to country, sex, and gestational age at birth. Sensitivity analyses using alternative outcome definitions. 10

Figure S3. Adjusted hazard ratios (HRs) of external causes of death according to country, sex, and gestational age at birth. Sensitivity analyses with additional adjustment for family history of suicide. 11

Figure S4. Unadjusted rates of death from external causes according to country, sex, and gestational age at birth. Sensitivity analysis in populations born 1987 or later. ... 12

Figure S5. Adjusted hazard ratios (HRs) of external causes of death according to country, sex, and gestational age at birth. Sensitivity analysis in populations born 1987 or later. ... 13

Figure S1. Flow chart showing inclusions and exclusions from the study population in each country.

Live born individuals recorded in the Medical Birth Registries
DK 1,476,052 FI 1,031,621 NO 2,075,809 SW 2,805,800

Exclusion: Death before 15 years
DK 13,799 (0.9%) FI 6,798 (0.7%) NO 24,021 (1.2%) SW 22,305 (0.8%)

Exclusion: Emigration before 15 years
DK 20,659 (1.4%) FI 6,423 (0.6%) NO 21,159 (1.0%) SW 95,899 (3.4%)

Individuals alive and living in the country of birth at age 15 years
DK 1,441,594 (97.7%) FI 1,018,400 (98.7%) NO 2,030,629 (97.8%) SW 2,687,596 (95.8%)

Exclusion: Missing gestational age
DK 69,302 (4.8%) FI 7,275 (0.7%) NO 121,147 (6.0%) SW 6,553 (0.2%)

Exclusion: Gestational age outside the range of 23-44 weeks
DK 108 (0.0%) FI 2 (0.0%) NO 7,689 (0.4%) SW 1,058 (0.0%)

Exclusion: Missing birthweight
DK 4,400 (0.3%) FI 274 (0.0%) NO 1,858 (0.1%) SW 7,260 (0.3%)

Exclusion: Implausible birthweight for gestational age[a]
DK 2,171 (0.2%) FI 2,792 (0.3%) NO 8,959 (0.4%) SW 5,890 (0.2%)

Exclusion: Missing information on sex, birth order or maternal age
DK 5,160 (0.4%) FI 1,474 (0.1%) NO 0 SW 0

Study populations
DK 1,360,453 (94.4%) FI 1,006,583 (98.8%) NO 1,890,826 (93.1%) SW 2,666,835 (99.2%)

Study populations born in 1987 or later
DK 929,470 (68.3%) FI 1,006,583 (100%) NO 848,313 (44.9%) SW 1,477,034 (55.4%)

[a] Defined as >+44% or <-66% from the intrauterine growth curves presented by Marsál K, Persson PH, Larsen T, Lilja H, Selbing A, Sultan B. Intrauterine growth curves based on ultrasonically estimated foetal weights. Acta Paediatr. Jul 1996;85(7):843-8. DK: Denmark, FI: Finland, NO: Norway, SW: Sweden. Percentages at each step are calculated from numbers in the previous inclusion box (blue) for each country.

Table S1. Overview of ICD versions and codes used to define outcomes, and when these were used in each country.

Table S1. Overview of ICD versions and codes used to define outcomes, and when these were used in each country.

	ICD-10	ICD-9	ICD-8
**Outcome of interest**			
All external causes of death	V01-Y89	E800-E999	E800-E999
Fatal transport accident	V01-V99, Y85	E800-E848, E929.0-1[a]	E800-E845, E940-E941
Suicide	X60-X84, Y87.0	E950-E959	E950-E959
Suicide including death of unknown intent	X60-X84, Y87.0 Y10-Y34, Y87.2	E950-E959, E980-E989	E950-E959, E980-E989
Death from drugs or alcohol[b]	E24.4, F10-F16, F18-F19, G31.2, G62.1, G72.1, I42.6, K29.2, K70, K86.0, O35.4-O35.5, P04.3-P04.4, Q86.0, T40.0-T40.3, T40.5-T40.9, T43.6, X40-X49, Y10-Y19	291, 303, 304, 571.0, 577, 860	291, 303, 304, 571.0, 577, 860
Death from illegal drugs or alcohol[c]	F10-F16, F18-F19, G31.2, G40.51, G62.1, G72.1, I42.6, K29.2, K70, K85.2- K85.3, K86.0, P04.3, Q86.0, X42, X45	291, 303, 304, 571.0, 577, 860	291, 303, 304, 571.0, 577, 860
**Period of use in Cause of Death Registry**			
Denmark	1994-	-	1970-1993
Finland	1996-	1987-1995	(1969-1986)
Norway	1996-	1986-1995	1969-1985
Sweden	1997-	1987-1996	1969-1986

[a] Not in use in Finland. [b] As defined by The Nordic Medico-Statistical Committee, excluding suicides. [c] Based partly on the definition from the Norwegian Institute of Public Health.

ICD: International Statistical Classification of Diseases and Related Health Problems

Table S2. Unadjusted and adjusted associations of gestational age at birth with mortality from all external causes during late adolescence and adulthood, by sex and country of birth.

Table S2. Unadjusted and adjusted associations of gestational age at birth with mortality from all external causes during late adolescence and adulthood, by sex and country of birth. Country-specific adjusted estimates are combined using a meta-analysis approach.

All external causes		Females				Males			
		Unadjusted		Adjusted [a]		Unadjusted		Adjusted [a]	
Country	Gestational age [b]	HR	95% CI	HR	95% CI	HR	95% CI	HR	95% CI
Denmark	23-33	1.94	1.14-3.30	1.88	1.10-3.19	1.19	0.86-1.63	1.17	0.85-1.61
	34-36	1.48	1.04-2.12	1.41	0.99-2.03	1.07	0.88-1.31	1.04	0.86-1.28
	37-38	1.01	0.80-1.29	0.99	0.78-1.26	1.05	0.94-1.18	1.06	0.94-1.19
	39-41	1		1		1		1	
	42-44	0.97	0.73-1.28	0.98	0.74-1.30	0.89	0.77-1.03	0.9	0.77-1.04
Finland	23-33	0.74	0.33-1.65	0.74	0.33-1.66	1.23	0.89-1.71	1.26	0.90-1.75
	34-36	0.93	0.63-1.39	0.93	0.62-1.38	1.08	0.88-1.31	1.09	0.89-1.33
	37-38	1.04	0.86-1.25	1.04	0.86-1.25	1.03	0.92-1.14	1.03	0.93-1.15
	39-41	1		1		1		1	
	42-44	0.89	0.61-1.29	0.89	0.61-1.29	1.05	0.86-1.28	1.06	0.87-1.30
Norway	23-33	1.5	1.09-2.07	1.5	1.09-2.06	1.07	0.89-1.30	1.08	0.90-1.31
	34-36	1.14	0.93-1.39	1.12	0.92-1.37	1.1	0.99-1.22	1.09	0.99-1.21
	37-38	1.13	1.00-1.27	1.13	1.00-1.27	1.03	0.97-1.10	1.04	0.98-1.10
	39-41	1		1		1		1	
	42-44	0.99	0.88-1.10	0.97	0.87-1.09	1.12	1.06-1.19	1.1	1.04-1.17
Sweden	23-33	1.7	1.28-2.25	1.66	1.25-2.20	1.09	0.90-1.31	1.06	0.88-1.29
	34-36	1.17	0.98-1.41	1.16	0.97-1.39	1.17	1.06-1.29	1.15	1.04-1.27
	37-38	1.06	0.96-1.17	1.04	0.94-1.15	1.11	1.05-1.18	1.1	1.04-1.16
	39-41	1		1		1		1	
	42-44	0.91	0.80-1.04	0.93	0.81-1.06	1.02	0.95-1.10	1.04	0.97-1.12
Combined	23-33			1.55	1.28-1.88			1.11	0.99-1.24
	34-36			1.15	1.02-1.29			1.11	1.04-1.18
	37-38			1.06	1.00-1.14			1.07	1.03-1.10
	39-41			1				1	
	42-44			0.95	0.88-1.03			1.06	1.02-1.11

[a] Adjusted for age, birth year, birth order, maternal age, and highest parental education at birth. For Swedish data, maternal education at birth was used as paternal education was not available. [b] Completed weeks.
CI: confidence interval, HR: hazard ratio.

Table S3. Unadjusted and adjusted associations of gestational age at birth with mortality from transport accidents during late adolescence and adulthood, by sex and country of birth.

Table S3. Unadjusted and adjusted associations of gestational age at birth with mortality from transport accidents during late adolescence and adulthood, by sex and country of birth. Country-specific adjusted estimates are combined using a meta-analysis approach.

Fatal transport accidents		Females				Males			
		Unadjusted		Adjusted [a]		Unadjusted		Adjusted [a]	
**Country**	**Gestational age [b]**	**HR**	**95% CI**	**HR**	**95% CI**	**HR**	**95% CI**	**HR**	**95% CI**
**Denmark**	23-33	1.58	0.65-3.84	1.6	0.66-3.89	1.28	0.80-2.04	1.27	0.80-2.04
	34-36	1.51	0.88-2.59	1.48	0.86-2.54	0.89	0.64-1.24	0.87	0.63-1.22
	37-38	1.02	0.72-1.46	1.04	0.73-1.48	1.11	0.93-1.31	1.13	0.95-1.34
	39-41	1		1		1		1	
	42-44	1.01	0.66-1.54	1.02	0.67-1.57	0.84	0.66-1.06	0.85	0.67-1.08
**Finland**	23-33	NE		NE		1.48	0.82-2.70	1.54	0.85-2.82
	34-36	0.95	0.39-2.32	0.94	0.38-2.30	1.02	0.68-1.55	1.04	0.69-1.57
	37-38	1.08	0.71-1.65	1.09	0.72-1.67	0.98	0.79-1.22	0.99	0.80-1.23
	39-41	1		1		1		1	
	42-44	1.72	0.92-3.19	1.69	0.90-3.18	0.8	0.51-1.25	0.8	0.51-1.25
**Norway**	23-33	1.46	0.78-2.73	1.55	0.83-2.90	1.09	0.78-1.54	1.17	0.83-1.64
	34-36	0.87	0.55-1.35	0.88	0.56-1.38	1.01	0.83-1.23	1.02	0.84-1.24
	37-38	1.02	0.80-1.29	1.05	0.83-1.34	0.95	0.85-1.07	0.97	0.86-1.09
	39-41	1		1		1		1	
	42-44	0.89	0.71-1.12	0.86	0.68-1.09	1.05	0.94-1.17	1.03	0.92-1.15
**Sweden**	23-33	0.95	0.43-2.13	0.99	0.44-2.22	1.14	0.78-1.67	1.18	0.81-1.73
	34-36	1.06	0.70-1.60	1.07	0.71-1.62	1.15	0.94-1.42	1.15	0.94-1.42
	37-38	1.03	0.83-1.29	1.07	0.86-1.33	1.05	0.93-1.18	1.07	0.95-1.20
	39-41	1		1		1		1	
	42-44	0.83	0.60-1.14	0.78	0.56-1.07	1.08	0.93-1.25	1.02	0.88-1.19
**Combined**	23-33			1.29	0.88-1.91			1.23	1.00-1.52
	34-36			1.07	0.83-1.38			1.05	0.92-1.18
	37-38			1.06	0.92-1.22			1.03	0.96-1.11
	39-41			1				1	
	42-44			0.9	0.76-1.07			0.99	0.91-1.08

[a] Adjusted for age, birth year, birth order, maternal age, and highest parental education at birth. For Swedish data, maternal education at birth was used as paternal education was not available. [b] Completed weeks.
CI: confidence interval, HR: hazard ratio, NE: Not estimated due to <5 deaths in this group.

Table S4. Unadjusted and adjusted associations of gestational age at birth with mortality from suicide during late adolescence and adulthood, by sex and country of birth.

Table S4. Unadjusted and adjusted associations of gestational age at birth with mortality from suicide during late adolescence and adulthood, by sex and country of birth. Country-specific adjusted estimates are combined using a meta-analysis approach.

Suicides		Females				Males			
		Unadjusted		Adjusted [a]		Unadjusted		Adjusted [a]	
Country	Gestational age [b]	HR	95% CI	HR	95% CI	HR	95% CI	HR	95% CI
Denmark	23-33	2.88	1.27-6.53	2.74	1.21-6.22	1.23	0.68-2.23	1.21	0.66-2.19
	34-36	1.11	0.52-2.37	1.06	0.49-2.29	1	0.67-1.48	0.98	0.66-1.46
	37-38	1.15	0.75-1.76	1.1	0.72-1.69	1	0.80-1.24	0.99	0.79-1.24
	39-41	1		1		0		1	
	42-44	1.09	0.67-1.79	1.12	0.69-1.84	1.1	0.85-1.43	1.12	0.87-1.45
Finland	23-33	1.22	0.51-2.96	1.23	0.51-2.99	0.84	0.46-1.53	0.87	0.48-1.58
	34-36	1.03	0.61-1.77	1.03	0.60-1.75	1.02	0.75-1.39	1.04	0.77-1.42
	37-38	1.01	0.77-1.33	1.01	0.77-1.32	0.99	0.84-1.16	0.99	0.85-1.17
	39-41	1		1		1		1	
	42-44	0.79	0.45-1.38	0.79	0.45-1.38	0.99	0.73-1.34	1.02	0.76-1.39
Norway	23-33	1.49	0.91-2.44	1.47	0.90-2.41	0.95	0.69-1.32	0.97	0.69-1.34
	34-36	1.24	0.92-1.66	1.22	0.91-1.64	1.08	0.91-1.28	1.08	0.91-1.28
	37-38	1.22	1.03-1.45	1.2	1.01-1.43	1.05	0.95-1.16	1.05	0.95-1.16
	39-41	1		1		1		1	
	42-44	0.99	0.83-1.17	1	0.84-1.18	1.21	1.10-1.32	1.2	1.09-1.32
Sweden	23-33	2	1.35-2.95	1.92	1.30-2.83	0.9	0.63-1.29	0.88	0.62-1.26
	34-36	1.13	0.86-1.48	1.11	0.85-1.46	1	0.83-1.19	0.98	0.82-1.18
	37-38	1.08	0.94-1.26	1.05	0.91-1.22	1.02	0.92-1.12	1	0.91-1.10
	39-41	1		1		1		1	
	42-44	0.86	0.70-1.05	0.9	0.74-1.11	1.05	0.93-1.18	1.1	0.97-1.24
Combined	23-33			1.76	1.34-2.32			0.95	0.77-1.17
	34-36			1.14	0.95-1.37			1.03	0.92-1.15
	37-38			1.1	0.99-1.21			1.02	0.96-1.08
	39-41			1				1	
	42-44			0.96	0.85-1.08			1.15	1.07-1.23

[a] Adjusted for age, birth year, birth order, maternal age, and highest parental education at birth. For Swedish data, maternal education at birth was used as paternal education was not available. [b] Completed weeks.
CI: confidence interval, HR: hazard ratio.

Table S5. Unadjusted and adjusted associations of gestational age at birth with mortality from drugs or alcohol during late adolescence and adulthood, by sex and country of birth.

Table S5. Unadjusted and adjusted associations of gestational age at birth with mortality from drugs or alcohol during late adolescence and adulthood, by sex and country of birth. Country-specific adjusted estimates are combined using a meta-analysis approach.

**Death from drugs or alcohol**		**Females**				**Males**			
		**Unadjusted**		**Adjusted [a]**		**Unadjusted**		**Adjusted [a]**	
**Country**	**Gestational age [b]**	**HR**	**95% CI**	**HR**	**95% CI**	**HR**	**95% CI**	**HR**	**95% CI**
	23-33	NE		NE		0.78	0.32-1.88	0.72	0.30-1.75
	34-36	1.49	0.65-3.41	1.32	0.58-3.01	1.27	0.84-1.91	1.17	0.77-1.77
**Denmark**	37-38	0.94	0.54-1.66	0.88	0.50-1.56	0.97	0.75-1.27	0.95	0.73-1.23
	39-41	1		1		1		1	
	42-44	0.75	0.37-1.55	0.76	0.37-1.58	0.85	0.60-1.19	0.84	0.60-1.18
	23-33	NE		NA		1.8	0.96-3.37	1.77	0.94-3.32
	34-36	NE		NE		1.44	0.96-2.15	1.43	0.96-2.13
**Finland**	37-38	1.02	0.64-1.65	1.01	0.63-1.63	1.14	0.91-1.44	1.15	0.91-1.45
	39-41	1		1		1		1	
	42-44	NE		NE		1.59	1.10-2.30	1.56	1.08-2.26
	23-33	1.19	0.59-2.39	1.12	0.56-2.25	1.3	0.92-1.82	1.24	0.88-1.74
	34-36	1.12	0.76-1.64	1.07	0.72-1.57	1.28	1.07-1.54	1.24	1.03-1.49
**Norway**	37-38	0.96	0.76-1.22	0.95	0.75-1.20	1.12	1.00-1.26	1.12	1.00-1.25
	39-41	1		1		1		1	
	42-44	1.1	0.90-1.35	1.06	0.87-1.30	1.21	1.08-1.35	1.16	1.04-1.29
	23-33	2.07	1.14-3.77	1.92	1.05-3.50	1.29	0.93-1.78	1.2	0.87-1.66
	34-36	1.72	1.22-2.43	1.65	1.16-2.33	1.39	1.18-1.64	1.34	1.13-1.58
**Sweden**	37-38	1.22	0.98-1.51	1.17	0.94-1.45	1.31	1.19-1.44	1.28	1.16-1.40
	39-41	1		1		1		1	
	42-44	1.09	0.83-1.44	1.16	0.88-1.52	0.98	0.86-1.12	1.04	0.91-1.19
	23-33			1.53	0.97-2.41			1.23	0.99-1.53
	34-36			1.35	1.07-1.71			1.29	1.16-1.45
**Combined**	37-38			1.05	0.90-1.21			1.18	1.10-1.26
	39-41			1				1	
	42-44			1.06	0.90-1.24			1.11	1.02-1.20

[a] Adjusted for age, birth year, birth order, maternal age, and highest parental education at birth. For Swedish data, maternal education at birth was used as paternal education was not available. [b] Completed weeks.
CI: confidence interval, HR: hazard ratio, NE: Not estimated due to <5 deaths in this group.

Table S6. Adjusted associations of gestational age with mortality from external causes, according to sex and country of birth. Sensitivity analyses using other outcome definitions for suicide and death from drugs or alcohol.

Table S6. Adjusted[a] associations of gestational age with mortality from external causes, according to sex and country of birth. Country-specific adjusted estimates are combined using a meta-analysis approach. Sensitivity analyses using other outcome definitions for suicide and death from drugs or alcohol.

Sex	Gestational age[b]	Denmark		Finland		Norway		Sweden		Combined	
		HR	95% CI	HR	95% CI	HR	95% CI	HR	95% CI	HR	95% CI
**Suicide, including unknown intent**											
	23-33	2.78	(1.30-5.94)	1.14	(0.47-2.77)	1.47	(0.89-2.40)	1.90	(1.33-2.70)	1.77	(1.37-2.29)
	34-36	1.05	(0.51-2.14)	0.95	(0.56-1.62)	1.21	(0.90-1.62)	1.13	(0.89-1.44)	1.13	(0.95-1.34)
Females	37-38	0.99	(0.66-1.50)	1.00	(0.78-1.30)	1.21	(1.02-1.44)	1.04	(0.91-1.19)	1.08	(0.98-1.19)
	39-41	1		1		1		1		1	
	42-44	1.19	(0.76-1.86)	0.73	(0.42-1.28)	1.00	(0.85-1.19)	0.92	(0.77-1.10)	0.97	(0.86-1.09)
	23-33	1.08	(0.59-1.96)	0.96	(0.55-1.66)	0.95	(0.69-1.33)	1.00	(0.75-1.33)	0.99	(0.81-1.19)
	34-36	1.04	(0.73-1.50)	1.06	(0.79-1.42)	1.08	(0.91-1.27)	1.04	(0.90-1.21)	1.06	(0.96-1.17)
Males	37-38	1.00	(0.81-1.23)	1.01	(0.87-1.18)	1.05	(0.95-1.16)	1.04	(0.96-1.13)	1.04	(0.98-1.10)
	39-41	1		1		1		1		1	
	42-44	1.08	(0.85-1.39)	1.08	(0.81-1.44)	1.20	(1.09-1.31)	1.06	(0.95-1.18)	1.13	(1.06-1.21)
**Death from illegal drugs or alcohol**											
	23-33	NE		NE		NE		2.53	(1.03-6.23)	NE	
	34-36	NE		1.01	(0.32-3.25)	0.97	(0.60-1.55)	1.92	(1.10-3.36)	1.26	(0.89-1.78)
Females	37-38	0.75	(0.34-1.67)	1.04	(0.04-0.30)	0.96	(0.73-1.27)	1.17	(0.81-1.69)	1.01	(0.83-1.24)
	39-41	1		1		1		1		1	
	42-44	NE		0.81	(0.26-2.57)	1.08	(0.85-1.36)	1.14	(0.71-1.84)	1.08	(0.88-1.33)

Table S6. Adjusted[a] associations of gestational age with mortality from external causes, according to sex and country of birth. Country-specific adjusted estimates are combined using a meta-analysis approach. Sensitivity analyses using other outcome definitions for suicide and death from drugs or alcohol.

Sex	Gestational age[b]	Denmark		Finland		Norway		Sweden		Combined	
		HR	95% CI	HR	95% CI	HR	95% CI	HR	95% CI	HR	95% CI
Males	23-33	NE		2.08	(1.07-4.06)	1.32	(0.91-1.91)	1.52	(0.98-2.37)	1.49	(1.14-1.93)
	34-36	0.94	(0.50-1.78)	1.08	(0.64-1.82)	1.26	(1.03-1.55)	1.42	(1.11-1.83)	1.28	(1.11-1.48)
	37-38	0.93	(0.64-1.36)	1.09	(0.83-1.43)	1.11	(0.97-1.26)	1.3	(1.12-1.50)	1.16	(1.06-1.27)
	39-41	1		1		1		1		1	
	42-44	1.02	(0.66-1.57)	1.73	(1.15-2.6)	1.17	(1.04-1.32)	1.16	(0.96-1.41)	1.19	(1.08-1.31)

[a] Adjusted for age, birth year, birth order, maternal age, and highest parental education at birth. For Swedish data, maternal education at birth was used as paternal education was not available. [b] Completed weeks

CI: confidence interval, HR: hazard ratio, NE: Not estimated due to <5 deaths in this group

Figure S2. Adjusted hazard ratios (HRs) of suicide and death from drugs or alcohol according to country, sex, and gestational age at birth. Sensitivity analyses using alternative outcome definitions.

Adjusted for age, birth year, birth order, maternal age, and highest parental education at birth. For Swedish data, maternal education at birth was used as paternal education was not available. HRs (squares or diamonds) with 95% confidence intervals (error bands). HRs for categories with <5 deaths are not shown. Confidence intervals expanding the range 0.44-4.0 are indicated by arrows. Note that the y-scales are logarithmic.

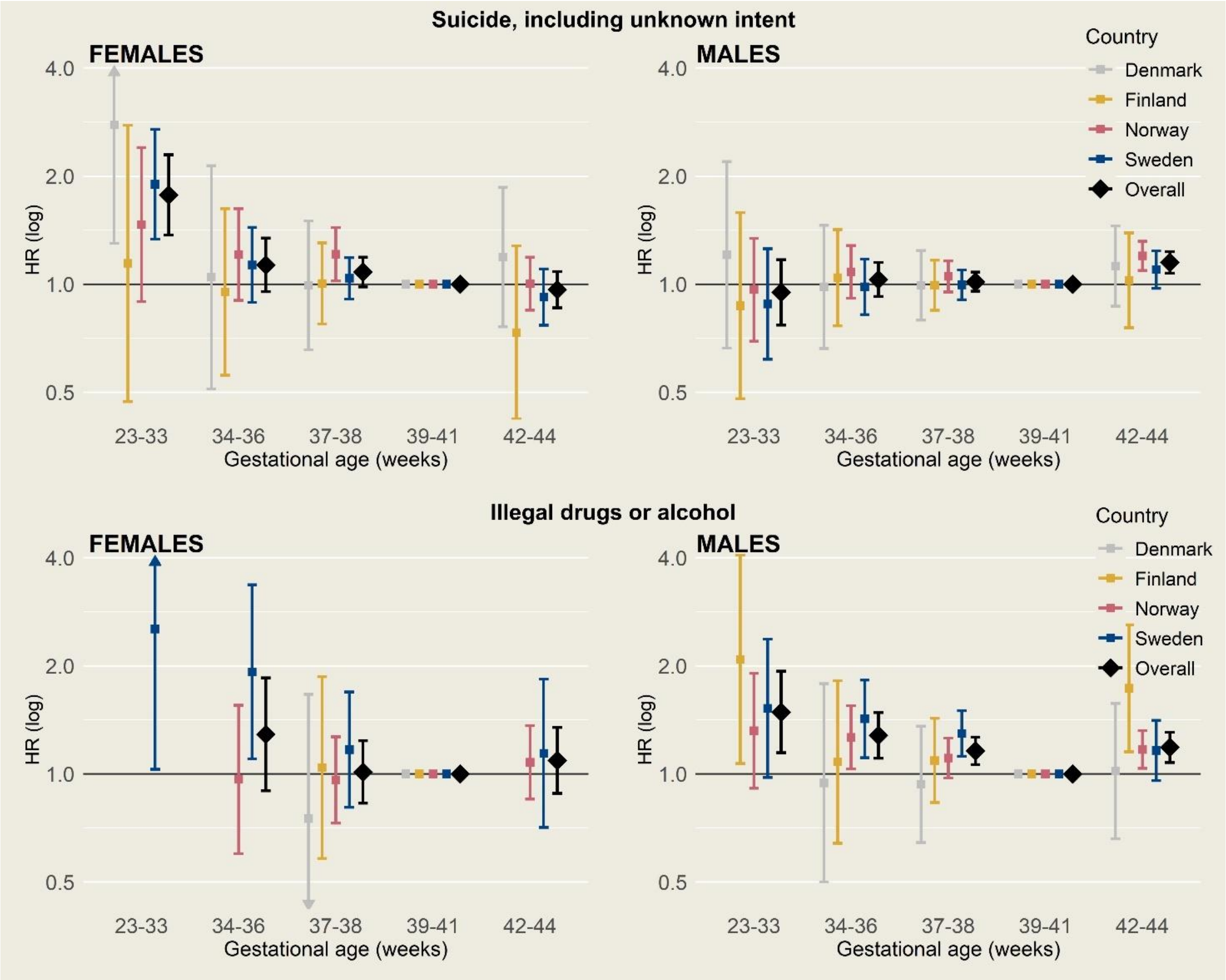

Figure S3. Adjusted hazard ratios (HRs) of external causes of death according to country, sex, and gestational age at birth. Sensitivity analyses with additional adjustment for family history of suicide.

Also adjusted for age, birth year, birth order, maternal age, and highest parental education at birth. For Swedish data, maternal education at birth was used as paternal education was not available. HRs (squares or diamonds) with 95% confidence intervals (error bands). HRs for categories with <5 deaths are not shown. Confidence intervals expanding the range 0.44-4.0 are indicated by arrows. Note that the y-scales are logarithmic.

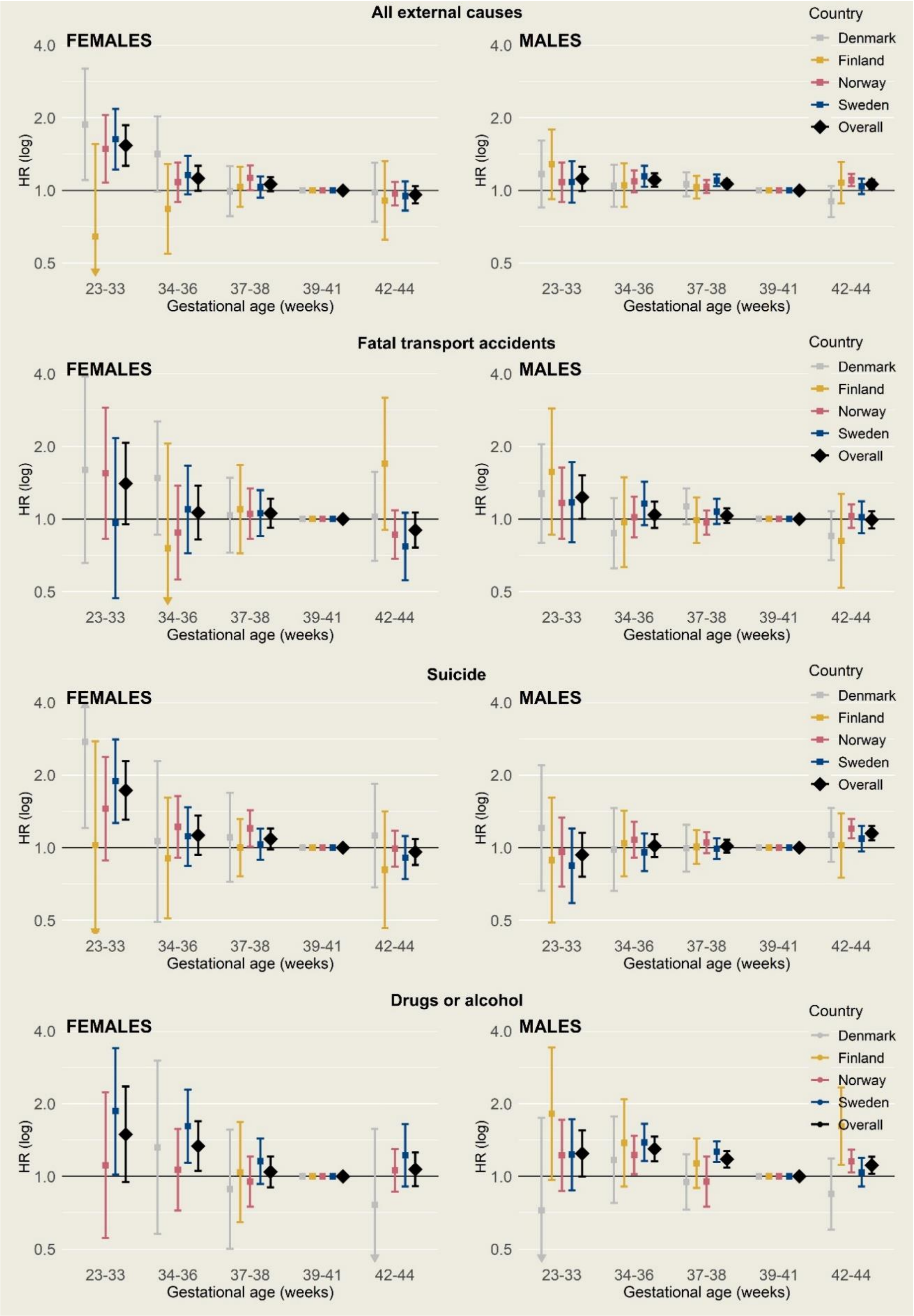

Figure S4. Unadjusted rates of death from external causes according to country, sex, and gestational age at birth. Sensitivity analysis in populations born 1987 or later.

Follow-up from age 15 years to a maximum of 50 years, among individuals born 1987-2001 in Denmark, 1987-2003 in Finland, 1987-2002 in Norway, and 1987-2001 in Sweden. Categories with no rates have either no events or are censored to maintain data privacy.

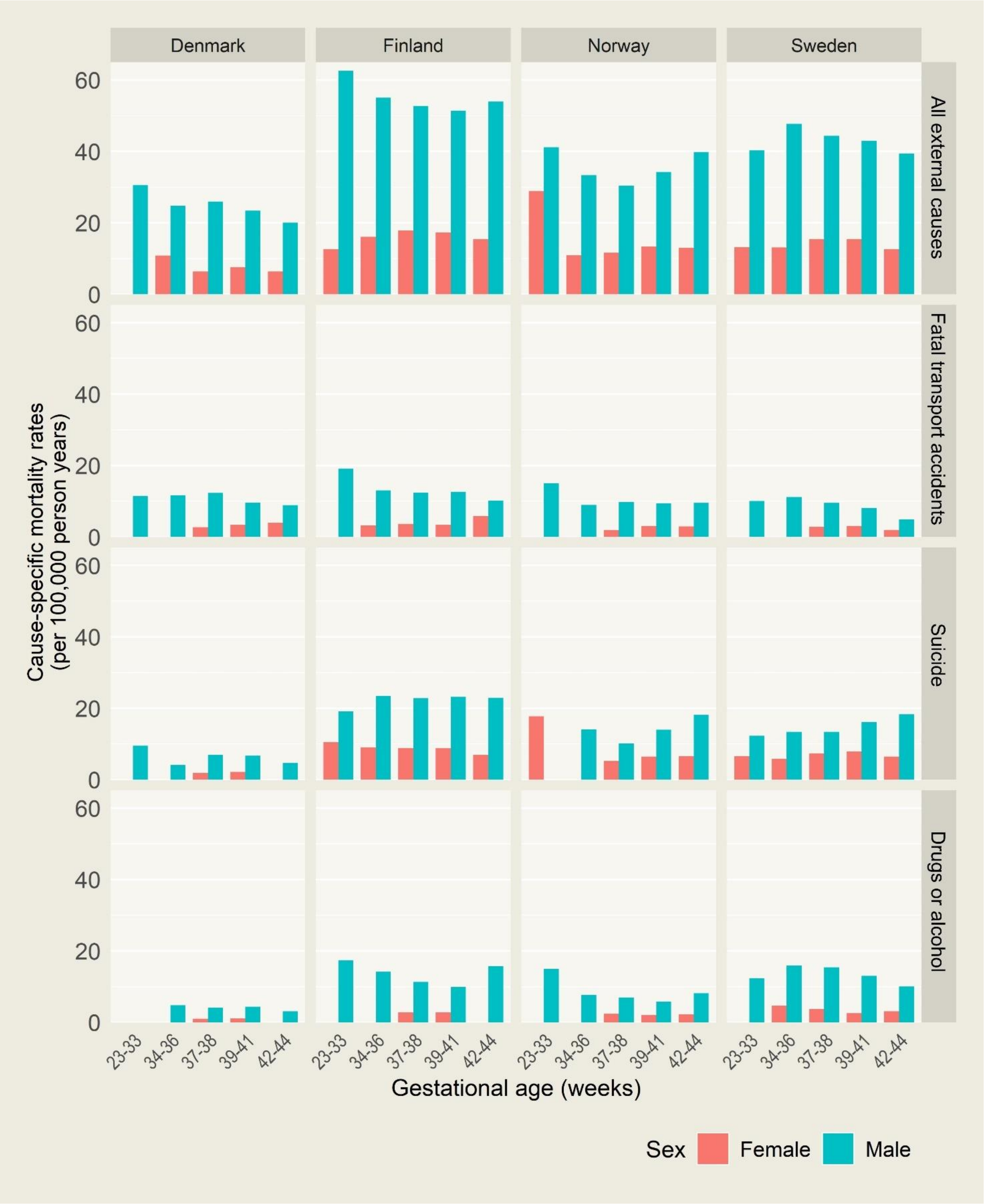

Figure S5. Adjusted hazard ratios (HRs) of external causes of death according to country, sex, and gestational age at birth. Sensitivity analysis in populations born 1987 or later.

Adjusted for age, birth year, birth order, maternal age, and highest parental education at birth. For Swedish data, maternal education at birth was used as paternal education was not available. HRs (squares or diamonds) with 95% confidence intervals (error bands). HRs for categories with <5 deaths are not shown. Confidence intervals expanding the range 0.44-4.0 are indicated by arrows. Note that the y-scales are logarithmic.

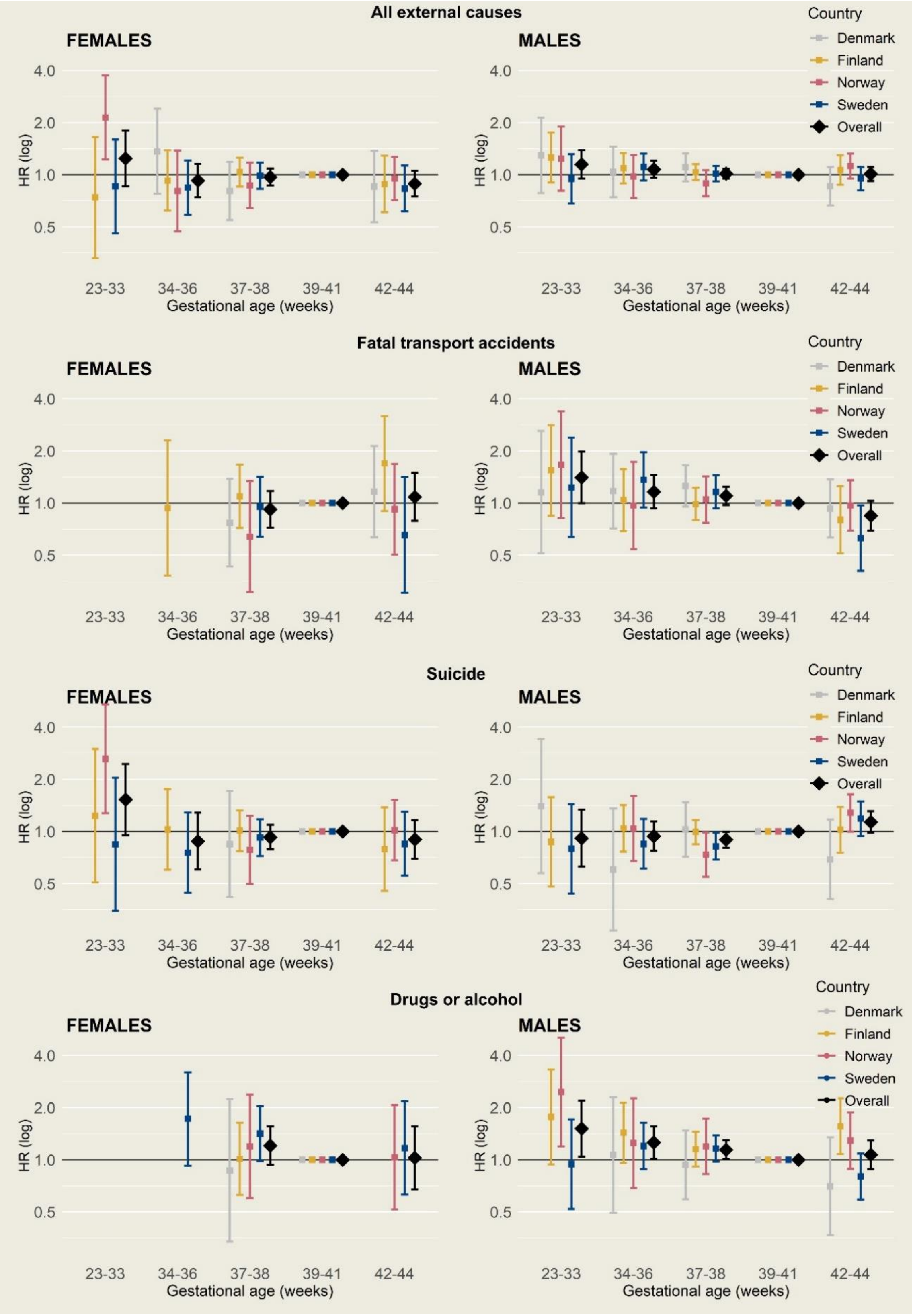

Supplement: Supplementary file 1 — Additional file 1. Fig. S1. Flow chart showing inclusions and exclusions from the study population in each country. Table S1. Overview of ICD versions and codes used to define outcomes, and when these were used in each country. Table S2. Unadjusted and adjusted associations of gestational age at birth with mortality from all external causes during late adolescence and adulthood, by sex and country of birth. Table S3. Unadjusted and adjusted associations of gestational age at birth with mortality from transport accidents during late adolescence and adulthood, by sex and country of birth. Table S4. Unadjusted and adjusted associations of gestational age at birth with mortality from suicide during late adolescence and adulthood, by sex and country of birth. Table S5. Unadjusted and adjusted associations of gestational age at birth with mortality from drugs or alcohol during late adolescence and adulthood, by sex and country of birth. Table S6. Adjusted associations of gestational age with mortality from external causes, according to sex and country of birth. Sensitivity analyses using other outcome definitions for suicide and death from drugs or alcohol. Fig. S2. Adjusted hazard ratios (HRs) of suicide and death from drugs or alcohol according to country, sex, and gestational age at birth. Sensitivity analyses using alternative outcome definitions. Fig. S3. Adjusted hazard ratios (HRs) of external causes of death according to country, sex, and gestational age at birth. Sensitivity analyses with additional adjustment for family history of suicide. Fig. S4. Unadjusted rates of death from external causes according to country, sex, and gestational age at birth. Sensitivity analysis in populations born 1987 or later. Fig. S5. Adjusted hazard ratios (HRs) of external causes of death according to country, sex, and gestational age at birth. Sensitivity analysis in populations born 1987 or later. [file 12916_2024_3731_MOESM1_ESM.pdf]
